# Paper-Based Bi-Material Cantilever Actuator Bending Behavior and Modeling

**DOI:** 10.3390/mi14050924

**Published:** 2023-04-25

**Authors:** Gordon Chen, Ashutosh Kumar, Hojat Heidari-Bafroui, Winfield Smith, Amer Charbaji, Nassim Rahmani, Constantine Anagnostopoulos, Mohammad Faghri

**Affiliations:** Microfluidics Laboratory, Department of Mechanical, Industrial and Systems Engineering, University of Rhode Island, 2 East Alumni Avenue, Kingston, RI 02881, USA

**Keywords:** paper-based sensor, bi-material cantilever, paper-based valve, bending response

## Abstract

In this paper, the behavior of the Bi-Material Cantilever (B-MaC) response deflection upon fluidic loading was experimentally studied and modeled for bilayer strips. A B-MaC consists of a strip of paper adhered to a strip of tape. When fluid is introduced, the paper expands while the tape does not, which causes the structure to bend due to strain mismatch, similar to the thermal loading of bi-metal thermostats. The main novelty of the paper-based bilayer cantilevers is the mechanical properties of two different types of material layers, a top layer of sensing paper and a bottom layer of actuating tape, to create a structure that can respond to moisture changes. When the sensing layer absorbs moisture, it causes the bilayer cantilever to bend or curl due to the differential swelling between the two layers. The portion of the paper strip that gets wet forms an arc, and as the fluid advances and fully wets the B-MaC, the entire B-MaC assumes the shape of the initial arc. This study showed that paper with higher hygroscopic expansion forms an arc with a smaller radius of curvature, whereas thicker tape with a higher Young’s modulus forms an arc with a larger radius of curvature. The results showed that the theoretical modeling could accurately predict the behavior of the bilayer strips. The significance of paper-based bilayer cantilevers lies in their potential applications in various fields, such as biomedicine, and environmental monitoring. In summary, the novelty and significance of paper-based bilayer cantilevers lie in their unique combination of sensing and actuating capabilities using a low-cost and environmentally friendly material.

## 1. Introduction

In its simplest form, a composite bilayer consists of two thin strips of different materials bonded together along their lengths. These bilayers have attracted a significant amount of attention over the years for their applications in the electronics [1,2], biomimetics [3], and biomedical [4,5] fields due to their versatility. One of their capabilities is self-actuation, where dimensional changes in the material layer(s) induced by external stimuli are transformed into controllable bending of the bilayer. This phenomenon is similar to bi-metallic thermostats, where two metallic strips with different coefficients of thermal expansion are bonded along their lengths. When the strips are subjected to a temperature change, the two materials expand or contract at different rates, resulting in bending actuation in the bonded pair.

The theory behind this behavior was first developed by Timoshenko in 1925 [6], who explored the bending of bi-metal strips subjected to uniform heating. Although Timoshenko’s analysis scope was limited to just metals and temperature, there have been a significant amount of simulations that show the bi-metal modeling can be applied to bilayers made of different materials and subjected to other external stimuli other than temperature. Materials such as polymers [7,8], hydrogels [9,10,11], and wood/fibers [12,13] have been extensively studied for their bending response when used in a bilayer configuration and exposed to various external stimuli such as light [14], electricity [15], pH levels [16], humidity/moisture content [17,18,19,20], or a combination of these factors. These studies demonstrate the potential of Timoshenko’s pioneering theory in describing the bending behavior of bilayers made of various materials subjected to various external stimuli, in addition to metallic bilayers and thermal changes.

This paper examines the behavior of the novel Bi-Material Cantilever (B-MaC), which is utilized in the microfluidic paper-based analytical device (μPAD) at the URI Microfluidics Lab [21]. The B-MaC is a simple bilayer consisting of a strip of paper laminated to a strip of tape that is designed to bend in response to fluid imbibition. Its intended application is to serve as a fluid transport mechanism to deliver multiple reagents in a sequential and timed manner autonomously within microfluidic assays [22]. In order to effectively integrate and optimize the B-MaC into such a device, it is important to understand its bending and tip deflection behavior when fluid is introduced to the device. For the purposes of this study, experiments were conducted using B-MaCs, fabricated from different papers and tapes, while their bending responses were observed and analyzed when the paper strip was wetted. The findings from the experiments were then used to develop an analytical model, which was modified to describe bending in response to hygroscopic stimuli. The results of these experiments were plotted and compared to the analytical model to verify the model’s ability to predict the bending of the B-MaCs.

Paper-based bilayer cantilevers have many potential applications as micro-machines due to their unique mechanical and physical properties. The paper-based B-MaCs can be used as sensors for detecting various types of chemical and biological substances. The cantilevers can be coated with a material that reacts with the substance being detected, causing a change in the cantilever’s mechanical properties that can be measured. Upon fluidic loading of the B-MaC, the unsupported tip can be made to bend. This property can be used in micro-actuators for various applications in microfluidics. The paper-based bilayer cantilevers can be used as energy harvesters, converting mechanical energy into electrical energy [23]. This can be useful in micro-power generation applications. The surface of the paper-based bilayer cantilever can be patterned with various materials or structures, making it useful for micro-patterning applications such as lithography. Overall, the paper-based bilayer cantilever has great potential as a micro-machine due to its low cost, simplicity, and versatility in various applications.

The reusability and longevity of a paper-based cantilever will depend on several factors, including the quality of the paper, the environment in which it is used, and the stress placed on the cantilever. Paper-based cantilevers are prone to bending, tearing, and other forms of damage, especially if they are exposed to moisture, heat, or other environmental factors that can weaken the paper fibers. Therefore, the reusability of a paper-based cantilever will depend on how much stress it is subjected to during its application and use. However, it’s expected that the bilayer cantilever wouldn’t be reused for most μPAD applications because of the horrification of the cellulose fibers after fluidic loading to be effective for reuse and the staining of the detection zone in colorimetric assays. Finally, while the durability and reusability of paper-based cantilevers may be limited, they are still a very cost-effective and environmentally friendly alternative to many other materials.

## 2. Experimental Section

### 2.1. B-MaC Concept of Design

**Figure 1:**
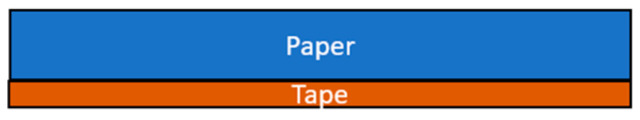
Illustration of a B-MaC (Not to scale).

The B-MaC investigated here is a bilayer consisting of a slender strip of paper and tape laminated together (Figure 1). Paper is a hygroscopic material that swells or shrinks when subjected to a change in moisture. Therefore, it is denoted as the active layer. Conversely, the tape is denoted as the passive layer since it does not experience dimensional changes in response to moisture. When the B-MaC is exposed to a fluid, the paper layer will increase in length because of the hygroscopic expansion of its cellulose fibers. The change in the length of the paper, however, is restricted due to the inextensibility of the tape. This develops an imbalance of internal forces within the B-MaC and causes the B-MaC to bend.

**Figure 2:**
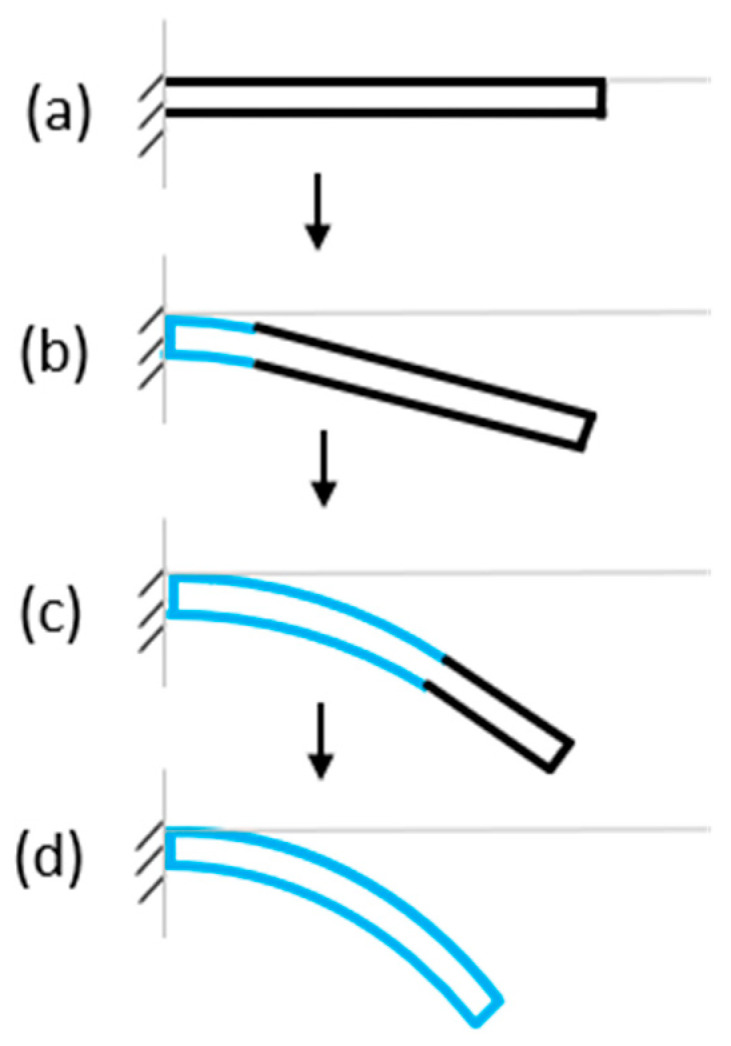
Sequential illustration of a B-MaC response to a fluid applied at the supported end. The B-MaC is oriented paper side up, tape side down. (**a**) Unloaded condition; (**b**) initial loading of fluid; (**c**) continued loading of fluid; (**d**) loaded condition.

When used in its intended application within a μPAD, the B-MaC acts as an actuator (unloaded condition) (Figure 2a). The B-MaC can then be activated by fluidic loading at the supported end. The B-MaC will immediately begin to bend at the wetted segment, thereby deflecting the free end of the B-MaC in the direction of the tape layer; see Figure 2b. The fluid front continues to wick along the length of the B-MaC, which sustains the bending along the wetted length, further deflecting the tip (Figure 2c). This process continues until the B-MaC is fully wetted (loaded condition) and the tip reaches a maximum deflection (Figure 2d).

### 2.2. Visual Experiments

The following experiments (Section 2.2.1, Section 2.2.2, Section 2.2.3 and Section 2.2.4) were carried out merely to observe the general bending behavior of a B-MaC when different paper orientations, paper types, tape types, and support locations are used. These experiments, however, were not used to obtain any measurable data. All the B-MaCs in these experiments were loaded by applying one drop of water at the support location, waiting until fully absorbed and wicked, then applying another drop, if necessary, until the entire length of the B-MaC was wetted.

#### 2.2.1. Paper Orientation

**Figure 3:**
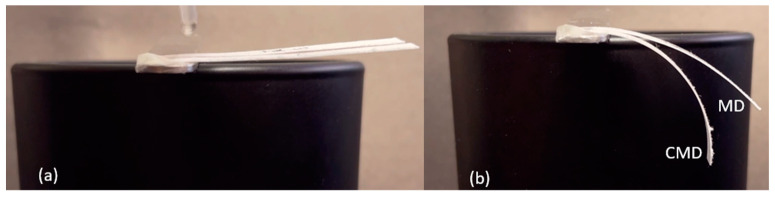
MD and CMD Comparison: (**a**) unloaded; (**b**) loaded. Larger curvature is achieved by the CMD B-MaC.

During the paper manufacturing process, the cellulose fibers tend to align their lengths in one direction, referred to as the machine direction (MD). When these individual fibers absorb water, they expand primarily in width but only slightly in length. Therefore, when the paper undergoes hygroscopic expansion, it will expand more in the cross-machine direction (CMD) compared to the MD. To verify this, two B-MaCs measuring 40 × 4 mm were fabricated with Whatman filter paper grade 41 (0.220 mm thick) and clear Scotch tape (0.058 mm thick). One had the paper oriented with the MD running in the length direction, while the other was oriented with the CMD running in the length direction. As seen in Figure 3, the experiment confirmed a noticeable difference in curvature between MD and CMD paper orientations, verifying the paper’s anisotropic properties. Greater curvature was achieved in the B-MaCs fabricated with their paper layer oriented in the CMD, signifying a positive correlation between paper expansion and B-MaC bending.

#### 2.2.2. Paper Type

**Figure 4:**
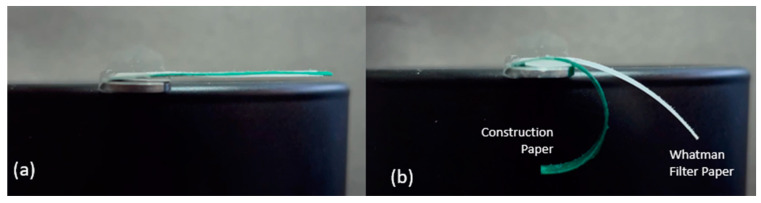
Comparison between paper types: (**a**) unloaded; (**b**) loaded. Larger curvature is achieved by the scrap (construction) paper B-MaC.

The effect of the paper type on the curvature of the B-MaC was also investigated in this study. Two B-MaCs measuring 30 × 4mm were fabricated, one with scrap paper 0.250 mm thick and the other with 0.220 mm thick Whatman filter paper grade 41. Both B-MaCs had the paper oriented in the CMD. Clear Scotch tape of 0.058 mm thickness was used as the passive layer. As seen in Figure 4, the green scrap paper resulted in a significantly larger curvature, suggesting that the paper type can have a significant effect on the bending characteristic of the B-Mac.

**Figure 5:**
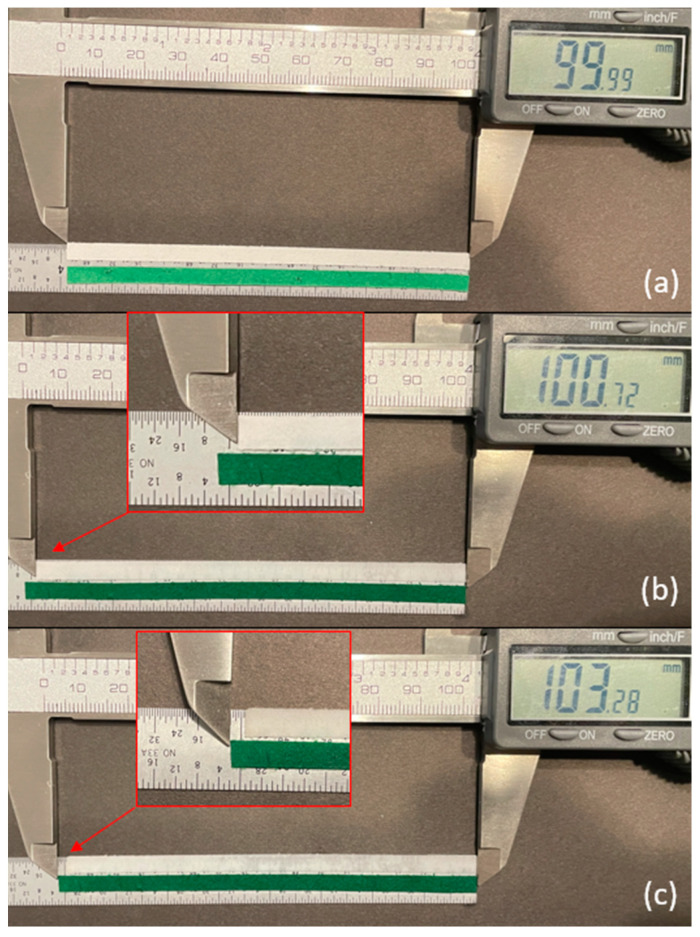
Hygroscopic expansion comparison between Whatman filter paper grade 41 and green scrap paper. (**a**) Both dry, ~100 mm; (**b**) saturated Whatman filter paper grade 41, ~100.7 mm; (**c**) saturated scrap paper, ~103.2 mm.

To determine the hygroscopic expansion of these two papers and to validate our hypothesis, a strip from each paper was cut, measuring 100 × 4 mm. Both strips were cut with the CMD of the paper in the direction of the strip length. In the dry condition, each strip measured 100 mm in length (Figure 5a). Drops of water were applied along the lengths until each strip was saturated. At full saturation, the Whatman paper expanded to 100.7 mm, representing a 0.7% expansion in length (Figure 5b). At full saturation, the green scrap paper expanded to 103.3 mm, representing a 3.3% expansion in length (Figure 5c). These results agree with the experiment above, thereby suggesting that larger hygroscopic expansion in the active layer results in greater B-MaC curvature.

#### 2.2.3. Tape Type

**Figure 6:**
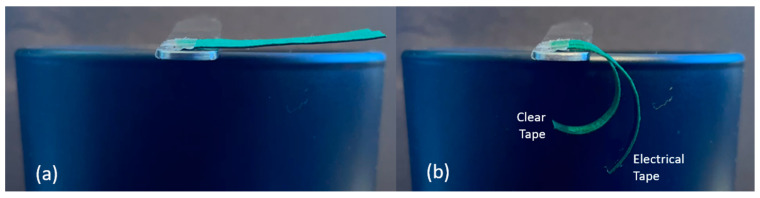
Comparison between tape types: (**a**) unloaded; (**b**) loaded. Smaller curvature is achieved by the electrical tape B-MaC.

Next, the effect of tape type on curvature was investigated. Two B-MaCs measuring 30 × 4 mm were fabricated, one with clear Scotch tape of 0.058 mm thickness and the other with industrial grade StikTek^TM^ electrical tape of 0.16 mm thickness. The 0.25-mm-thick scrap paper in CMD orientation was used for both B-MaCs. As seen in Figure 6, the electrical tape resulted in a smaller curvature, suggesting that thicker, more rigid tapes decrease the bending of a B-MaC.

#### 2.2.4. B-MaC Length, Support Location, Orientation

**Figure 7:**
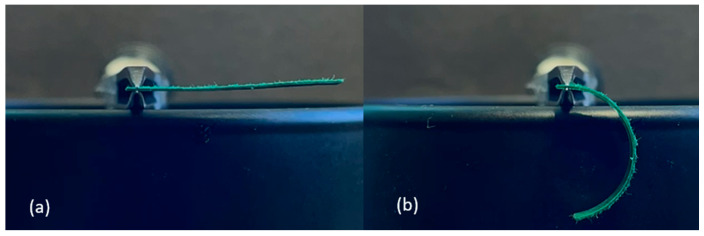
Cantilever supported 23 × 4 mm B-MaC. (**a**) Unloaded; (**b**) loaded. B-MaC bends into a semicircle.

**Figure 8:**
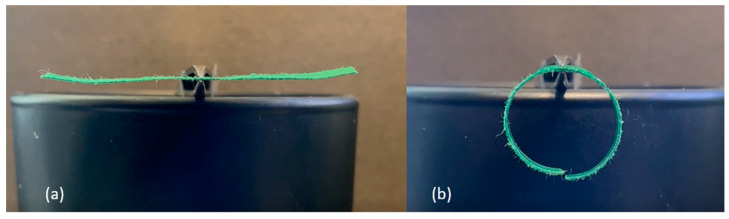
Center-supported 46 × 4 mm B-MaC. (**a**) Unloaded; (**b**) loaded. B-MaC bends into a near-perfect circle.

**Figure 9:**
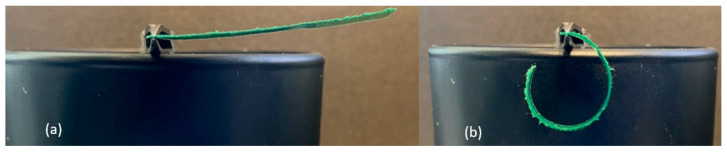
Cantilever supported 46 × 4 mm B-MaC. (**a**) Unloaded; (**b**) loaded. B-MaC bends into an oval.

**Figure 10:**
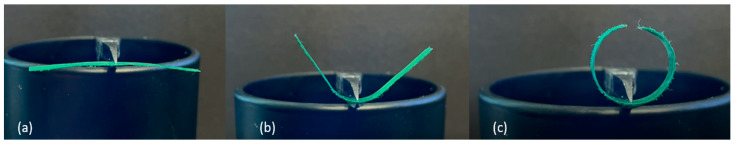
Inverted center-supported 46 × 4 mm B-MaC. (**a**) Unloaded; (**b**) half the length of B-MaC is fully wetted; (**c**) loaded. B-MaC bends upwards into a near perfect circle.

**Figure 11:**
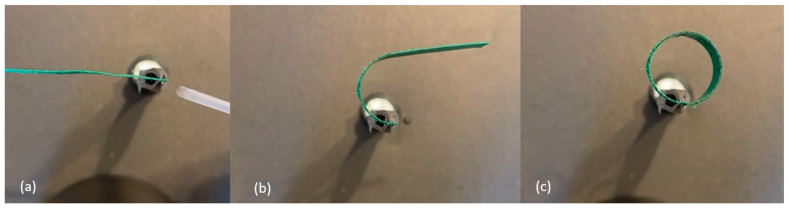
Vertically supported 46 × 4 mm B-MaC to negate bending effects due to self-weight (bending perpendicular to gravity). (**a**) Unloaded; (**b**) half the length of B-MaC is fully wetted; (**c**) loaded. B-MaC bends into a perfect circle.

The following experiments were conducted to determine the effects of overall length, support location, and orientation of the passive-active layer. All B-MaCs were fabricated from green scrap paper (0.25 mm thick), with the CMD oriented in the direction of the strip length, and clear Scotch tape (0.058 mm thick). The B-MaCs fabricated using this paper-tape combination bent into a semicircle at full saturation when the total length was 23 mm (Figure 7). At 46 mm in length, the B-MaC bent into a near-perfect circle when supported at the center, as seen in Figure 8. However, when supported as a cantilever, the 46 mm long B-MaC was not able to bend into a circle and instead bent into a spiral, as shown in Figure 9. This is likely a result of the weight of the B-MaC pulling it down, because when the B-MaC was flipped with the tape side facing up, it bent upward into a near-perfect circle, as seen in Figure 10. The last visual experiment eliminated the bending effects of gravity by orienting the B-MaC on vertical support to ensure gravity was not acting in the same plane as bending. As seen in Figure 11, the 46 mm-long B-MaC deformed into a perfect circle at full saturation. This suggests that the hygroscopic expansion produces constant uniform bending and confirms that a B-MaC will form into a full circle.

### 2.3. Laboratory Experiment

**Figure 12:**
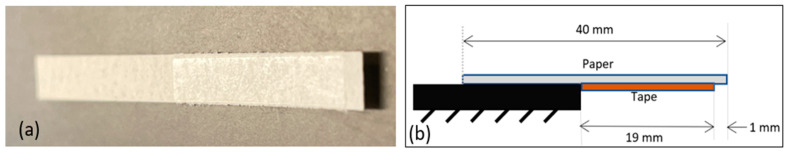
(**a**) Whatman 41 filter paper B-MaC; (**b**) dimensional illustration of the B-MaC.

**Figure 13:**
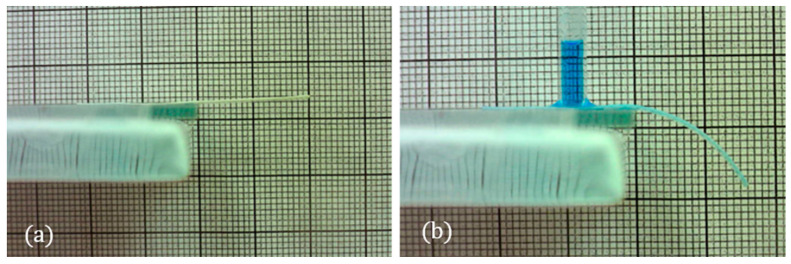
Test setup: (**a**) tape side down B-MaC positioned on the fixture in the unloaded condition; (**b**) actuated B-MaC in the loaded condition.

The following experiment was performed in the URI Microfluidics Laboratory to capture measurable actuation data of an optimized B-MaC currently under development for use in a μPAD. The B-MaC is fabricated from a cross-machine direction 40 × 4 × 0.22 mm-strip of Whatman filter paper grade 41 bonded to a 19 × 4 × 0.058 mm strip of Scotch Tape 600. The 20 mm non-taped segment of paper functions as both the support end for the cantilever and the fluid loading area (Figure 12b). The B-MaC was secured to a fixture and activated by applying blue-dyed water (Figure 13). In this experiment, a capillary tube with a static pressure head was utilized to apply the fluid continuously and evenly. Additional details concerning the materials, test conditions, test procedure, and equipment can be found in [24].

A total of four B-MaCs were actuated with the tape side oriented down for downward deflection. Video recordings of the four runs were captured and subsequently reviewed to visually measure the curvature and tip deflection utilizing the background grid. The curvature was determined by measuring the discrete x and y coordinates of the B-MaC after full actuation. Tip deflection was determined by measuring the discrete vertical tip position each time the fluid wicked 2 mm along the length of the B-MaC. These data points were plotted and used to gauge the accuracy of the proposed analytical model. Plots and discussions will be presented in the results sections.

## 3. Theoretical Model

In order to model the B-MaC when it is subjected to fluidic actuation, two sections have to be considered, namely, the wetted length *(L_wet_*) and the dry length (*L_dr_*_y_). The wetted length was modeled utilizing the hygroscopic effect, and the dry length remains unaffected by fluid. This approach can determine the curvature of the wetted length and its suitability for bilayers such as a B-MaC. The dry length was modeled as a tangent line originating from the end of the wetted length and was formulated using arc length relationships. Coupling the wetted length and dry length models together provided a final analytical model to describe the actuation behavior of a B-MaC.

The behavior of response deflection in a B-MaC is an intricate problem involving the structural deformation upon fluidic loading of the bilayer upon wetting. Hence, it is a coupled fluid-structure problem. To approach a simpler model, the fluid and structure are decoupled and studied under the following assumptions:

- Static Deformation: The response deflection of a B-MaC is the sole function of wetted length, and time dependence is not considered.
- The fluidic loading is determined by the wetted length of the bilayer, governed by the Lucas-Washburn equation.
- The cross-section of the bilayer remains normal to the neutral axis before and after deformation.
- The thickness of the bilayer is negligible in comparison to the radius of curvature.

The velocity and direction of fluid flow in paper-based cantilevers depend on the specific design of the cantilever and the properties of the fluid being analyzed. These factors are not typically directly measured for paper-based cantilevers. However, the cantilevers can be used to indirectly detect changes in fluid flow by detecting changes in humidity or other environmental factors that may be indicative of fluid flow. This paper considers a static model of a bilayer with dry and wet lengths. The fluid flow in filter paper is governed by the Lucas-Washburn equation, where the wetted length is proportional to the square root of the time for the wetting.

### 3.1. Wetted Length

**Figure 14:**
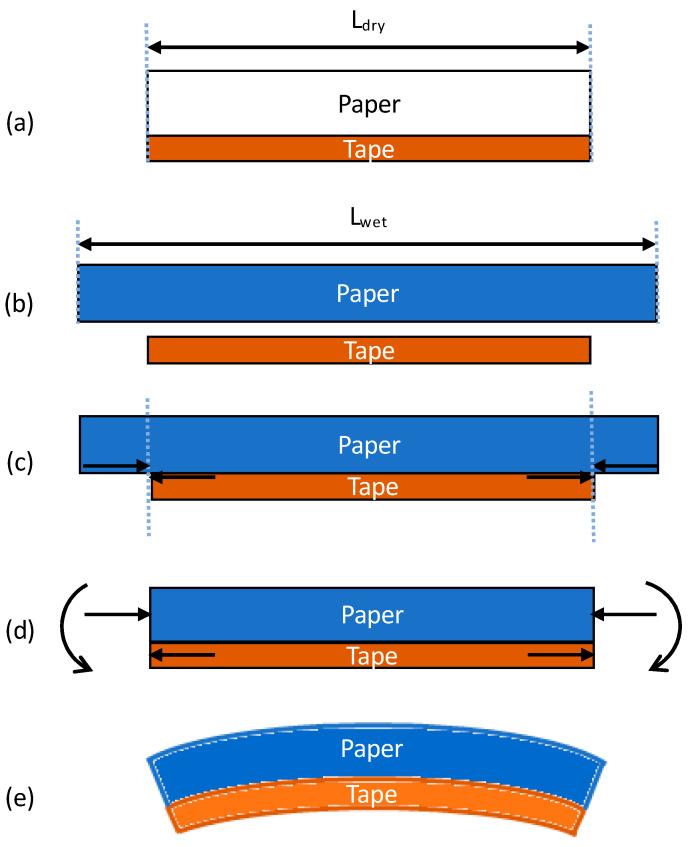
Sequential illustration for B-MaC bending (**a**) B-MaC in neutral (dry) condition; (**b**) B-MaC in unboned condition with paper undergoing hygro-expansion; (**c**) B-MaC in boned condition with paper undergoing hygro-expansion; (**d**) B-MaC in bonded condition experiencing tension and compression force due to hygro-expansion; (**e**) B-MaC in bonded condition experiencing bending.

**Figure 15:**
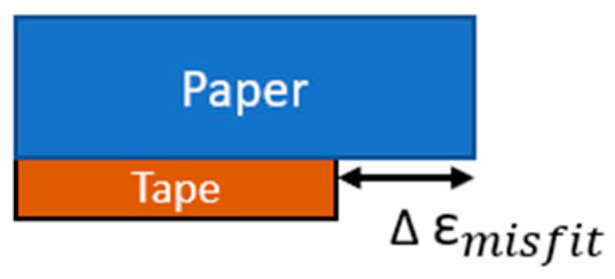
Misfit strain from hygroscopic expansion.

Let us consider the hygroscopic expansive bending process within the B-MaC, as illustrated in Figure 14. (a) Before fluid is introduced, the B-MaC is in the neutral unloaded (dry) condition, and the paper and tape are in equilibrium with no internal or external stresses present. (b) If the two layers are unbonded and fluid is introduced, the paper will experience stress-free, pure hygroscopic expansion and lengthen relative to the tape (for simplicity, only in-plane (length) direction expansion is considered). This difference in length is the strain mismatch, or misfit strain, which is defined as the difference between the stress-free dimensions of two or more constituents that are bonded together [25] (Figure 15). (c) Since the paper and tape are in fact bonded together, the lengthening of the paper can be accommodated by the structure forming an arc. The misfit strain generates a tensile force on the tape, which is balanced by an equal and opposite compressive force on the paper. (d) These forces act along the bearing surface but can be equivalently drawn at the centroid of each layer, accompanied by the end moment required for complete static equilibrium. (e) The end moments will generate out-of-plane concave-down curvature of the B-MaC (bending towards the tape layer) to balance the forces within the B-MaC.

**Figure 16:**
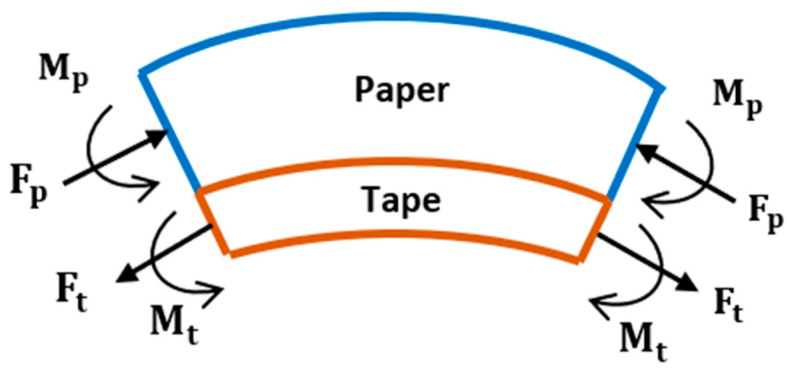
Free body diagram of a small, wetted length section of B-MaC (not to scale).

Based on the observations from the visual experiments performed and the fact that the cross-sectional area along the length of the B-MaC is constant, it is assumed that the hygroscopic expansion at the moving fluid front produces a constant force resulting in constant curvature. Considering a small element for the wetted length of the B-MaC (Figure 16), the force and moment balance is described below.

When the paper swells during imbibition, a misfit strain is generated, resulting in an axial tensile force *F_t_* on the tape’s cross-section. Since there are no external forces applied, static equilibrium requires an equal and opposite compressive force *F_p_* on the paper’s cross-section:

(1) $$F_{t}=F_{p}=F$$

**Figure 17:**
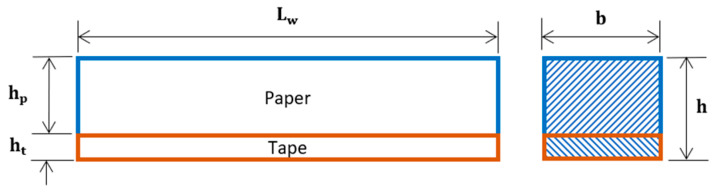
Schematic illustration of dimensional parameters (not to scale).

Similarly, the bending moments in both layers must be in static equilibrium with the axial forces. Using the dimensional parameters in Figure 17 and performing moment equilibrium balance at the bearing surface to equate forces and moments results in:

$$M_{p}+M_{t}=F_{p}\left(\frac{1}{2}h_{p}\right)+F_{t}(\frac{1}{2}h_{t})$$

(2) $$M_{p}+M_{t}=F\frac{\left(h_{p}+h_{t}\right)}{2}$$

Assuming the wetted paper and tape behave as linear elastic materials, for constant Young’s modulus and geometrical parameters, we can apply the following relationships to the bilayers:

(3) $$M_{p}=\frac{E_{p}I_{p}}{\rho };\;M_{t}=\frac{E_{t}I_{t}}{\rho }\;\mathrm{and}\;I_{p}=\frac{bh_{p}{}^{3}}{12};\;I_{t}=\frac{bh_{t}{}^{3}}{12}$$

where *E_p_* and *E_t_* are Young’s moduli of the paper and tape, respectively; *I_p_* and *I_t_* are the area moments of inertia for paper and tape, respectively; *ρ* is the radius of curvature; *b* is the width of the actuator; and *h_p_* and *h_t_* are the thickness of the paper and tape, respectively.

Substituting $M_{p}$ and $M_{t}$ from (3) into the moment equilibrium Equation (2) yields:

(4) $$\frac{\;E_{p}\;I_{p}}{\rho }+\frac{\;E_{t}\;I_{t}}{\rho }=F\frac{\left(h_{p}+h_{t}\right)}{2}\;$$

Equation (4) has two unknown variables: *F* and *ρ*. Therefore, another equation is required to solve for these variables. Assuming the two layers are sufficiently bonded and that plane sections in the cross-section remain planar after bending, one can conclude that the total strains at the bearing surface must be equal. Therefore, strain compatibility at the bearing surface can be applied so that the total strain in the paper is equal to the total strain in the tape:

(5) $$\varepsilon_{p\;total}=\varepsilon_{t\;total}$$

When the B-MaC is fluidically loaded, the total strain in the paper layer is the sum of the elastic and hygroscopic misfit strains. The total strain in the tape layer is simply the elastic strain:

(6) $$\varepsilon_{p\;elastic}+\varepsilon_{misfit}=\varepsilon_{t\;elastic}$$

The elastic strains in both layers can be decomposed into a sum of elongation strain and bending strain:

(7) $$\frac{F}{\;E_{p}h_{p}b}+\frac{h_{p}}{2\rho }+\varepsilon_{misfit}=-\frac{F}{\;E_{t}h_{t}b}-\frac{h_{t}}{2\rho }$$

Rearranging:

(8) $$\frac{1}{2\rho }\left(h_{p}+h_{t}\right)=-\varepsilon_{misfit}-\left(\frac{1}{\;E_{p}h_{p}b}+\frac{1}{\;E_{t}h_{t}b}\right)F$$

Substituting *F* from Equation (4) into Equation (8) yields:

(9) $$\frac{1}{2\rho }\left(h_{p}+h_{t}\right)=-\varepsilon_{misfit}-\frac{2}{\rho }\left(\frac{1}{\;E_{p}h_{p}b}+\frac{1}{\;E_{t}h_{t}b}\right)\left(\frac{\;E_{p}\;I_{p}+E_{t}\;I_{t}}{h_{p}+h_{t}}\right)$$

Solving for *ρ* [26]:

(10) $$\rho =\frac{\;E_{t}{}^{2}\;h_{t}{}^{4}+4\;E_{t}\;E_{p}\;h_{t}{}^{3}h_{p}+6\;E_{t}E_{p}\;h_{t}{}^{2}\;h_{p}{}^{2}+4\;E_{t}\;E_{p}h_{t}\;h_{p}{}^{3}+E_{p}{}^{2}\;h_{p}{}^{4}}{6(-\varepsilon_{misfit})(\;E_{p}\;E_{t})(h_{p}h_{t})(h_{p}+h_{t})}$$

(11) $$\rho =\frac{h\left(3{\left(1+m\right)}^{2}+\left(1+mn\right)\left(m^{2}+\frac{1}{mn}\right)\right)}{6\left(-\varepsilon_{misfit}\right){\left(1+m\right)}^{2}\;}$$

where:

(12) $$m=\frac{\;h_{t}}{\;h_{p}}\;\;;\;\;\;n=\frac{\;E_{t}}{\;E_{p}}\;\;;\;\;\;h=\;h_{t}+h_{p}\;\;;\;-\varepsilon_{misfit}=\varepsilon_{hygro}=\frac{\;L_{sat}-L_{dry}}{\;L_{dry}}$$

Figure 14 defines *L_sat_* and *L_dry_* as the length of paper layer utilized to construct B-MaC at full saturation and in the dry state, respectively. The negative misfit strain in Equation (11) signifies the concave curvature of the B-MaC towards the tape layer. This is the governing equation to determine the radius of curvature for the wetted length of a B-MaC. From this equation, it can be seen that the radius is dependent on the misfit strain, Young’s modulus ratio, thickness ratio, and thickness of the composite layer. Equation (10) considers Young’s modulus of the wet paper, and tape, being hydrophobic in nature, will not wet upon fluid loading; therefore, tape modulus will remain the same for the dry or wet state. This formulation considers the static deflection of the bilayer cantilever; therefore, the curvature is attained when the paper and tape layer of the B-MaC is fully saturated when loaded with fluid.

**Figure 18:**
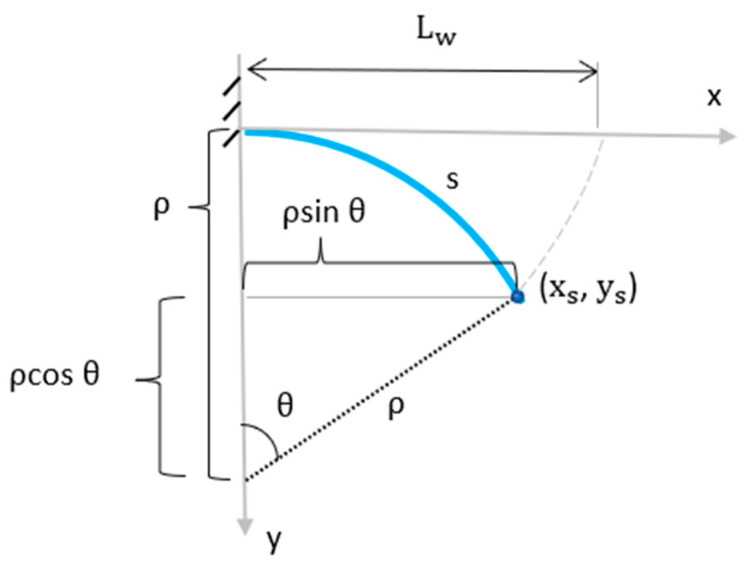
Schematic of the B-MaC wetted length.

Once the radius of curvature is determined, the location of the wetted length in the cartesian coordinate system has to be determined when the B-MaC is supported as a cantilever. As seen in Figure 18, the arc denoted s represents the wetted length of the B-MaC, and the curvilinear points *x_s_* and *y_s_* represent the coordinates for the wetted fluid front.

Using the arc length formula and trigonometry, the curvilinear points *x_s_* and *y_s_* can be determined as follows:

(13) $$\rho =\frac{s}{\theta }$$

(14) $$x_{s}=\rho \sin\theta$$

(15) $$y_{s}=\rho \left(cos\theta -1\right)$$

Knowing that *s = L_w_* due to the inextensibility of the tape, *θ* is determined to be:

(16) $$\theta \;=\frac{L_{w}}{\rho }$$

Substituting (16) into Equations (14) and (15) yields:

(17) $$x_{s\;}=\rho \;sin\left(\frac{L_{w}}{\rho }\right)$$

(18) $$y_{s}=\rho \left(cos\left(\frac{L_{w}}{\rho }\right)-1\right)$$

Equations (17) and (18) represent the cartesian coordinates of the wetted length and are functions of variables *L_w_* and *ρ*.

### 3.2. Dry Length

**Figure 19:**
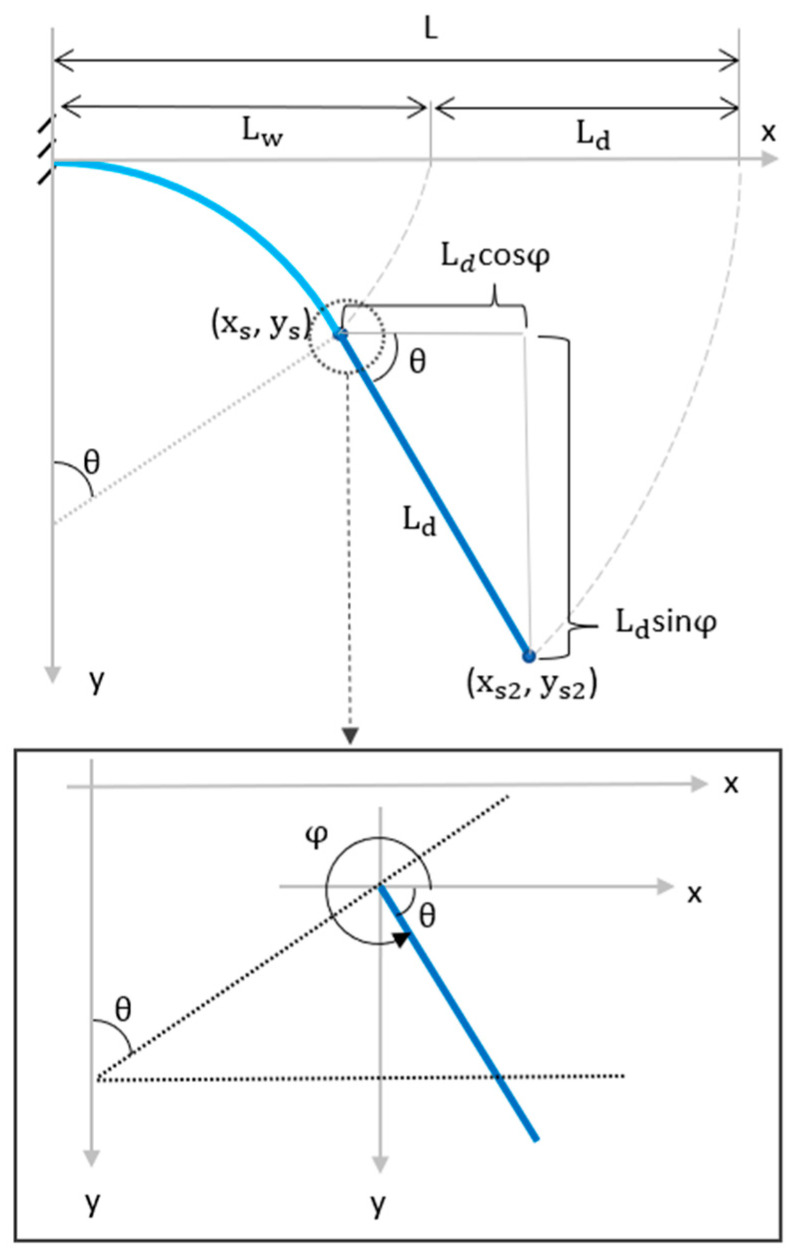
Schematic of the B-MaC dry length.

The dry length is the portion of the B-MaC that the wicking fluid front has not yet reached. Since it is not wet, it remains straight. As seen in Figure 19, the dry length is the difference between the B-MaC total length and the wetted length:

(19) $$L_{d}=L-L_{w}$$

Its orientation will be tangent to the arc at the wetted length tips *x_s_* and *y_s_*; therefore, its orientation angle will be:

(20) φ=2π−θ 

Using trigonometry, the dry length tip coordinates *x_s*2*_* and *y_s*2*_* are as follows:

(21) $$x_{s2}=x_{s}+L_{d}cos\varphi$$

(22) $$y_{s2}=y_{s}+L_{d}sin\varphi$$

Substituting (17)–(19) into (21) and (22) yields:

(23) $$x_{s2}=\rho \;sin\left(\frac{L_{w}}{\rho }\right)+\left(L-L_{w}\right)cos\left(2\pi -\frac{L_{w}}{\rho }\right)$$

(24) $$y_{s2}=\rho \left(cos\left(\frac{L_{w}}{\rho }\right)-1\right)+\left(L-L_{w}\right)sin\left(2\pi -\frac{L_{w}}{\rho }\right)$$

Equations (23) and (24) represent the dry length tip and are functions of variables *L*, *L_w_*, and *ρ*.

### 3.3. Final Combined Model

Utilizing Equations (11), (12), (17), (18), (23) and (24) provides the final analytical model to determine the actuation behavior of a B-MaC. Equation (11) determines the radius of curvature as a function of the layer height ratio (m), Young’s moduli ratio (n), total height (h), and misfit strain (*ε_misfit_*) parameters (12). The radius can then be inserted into Equations (17) and (18) to determine the curvilinear position of the fluid front as a function of the wetted length, or into Equations (18) and (19) to determine the position of the B-MaC tip as a function of the wetted length.

## 4. Results and Discussion

### 4.1. Analytical Model Plots

The analytical model was plotted using Matlab software (version) with the following parameters that describe the B-MaC (Whatman filter paper grade 41 and Scotch tape) from Section 2.3: *L* = 20 mm; ε_hygro_ = 0.007; *E_p_* = 24 MPa; *E_t_* = 150 MPa; *h_p_* = 0.220 mm; *h_t_* = 0.058 mm. The hygroscopic expansion parameter, *ε_hygro_* = 0.007, was determined from the visual experiment in Section 2.2.2. Young’s modulus for saturated paper, *E_p_* = 24 MPa, was taken from the literature [27], where saturated Whatman filter paper grade 1 was used. Young’s modulus for tape, *E_t_* = 150 MPa, was determined from tensile tests performed at the URI Microfluidics Lab. The saturated paper height (thickness), *h_p_* = 0.220 mm, is assumed to be equivalent to the dry paper height, assuming there is negligible swelling in the thickness direction.

**Figure 20:**
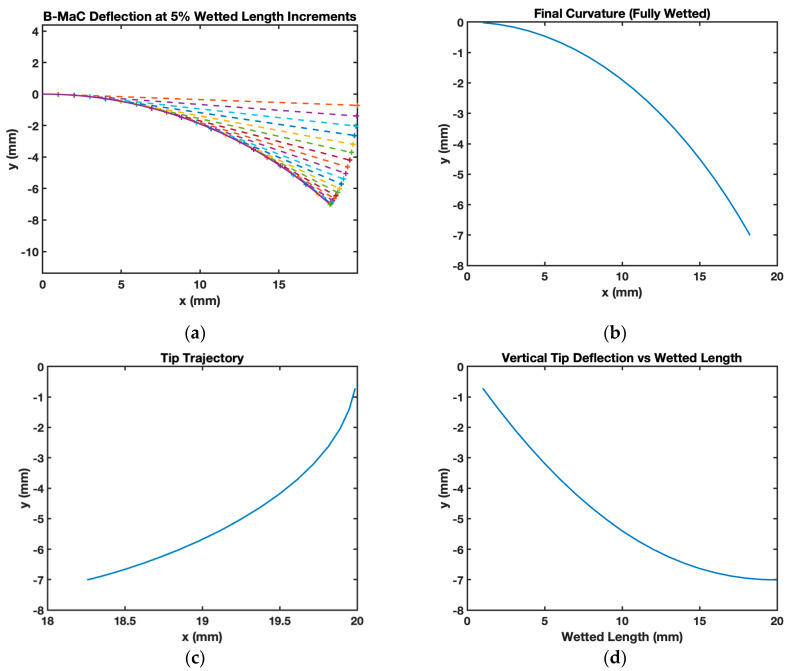
Matlab plots for the analytical model of a Whatman filter paper grade 41 Scotch tape B-MaC. (**a**) The B-MaC wetted curvature and corresponding dry length for every 5% wetted length increment; (**b**) final curvature after full actuation; (**c**) tip trajectory after full actuation; (**d**) vertical tip deflection as a function of wetted length.

The curvature of each incremental element of the B-MaC is obtained by substituting the parameter mentioned above in Equation (11). The curvature for the overall wetted length of the B-MaC is then obtained step by step for all the elements, as shown in Figure 20. The model predicted that a B-MaC with the given dimensions had a 27.28 mm radius of curvature and a 7 mm maximum vertical tip deflection. Figure 20a plots the bending-deflection sequence of the B-MaC as the wetted length increases by 5% increments, represented by the changing colors. The asterisks along the curvature represent the wetted length trajectory, which is plotted as a continuous curve in Figure 20b. The corresponding-colored asterisks at the end of the tangent line in Figure 20a represent the dry tip trajectory, which is plotted as a continuous curve in Figure 20c. Lastly, a plot of the B-MaCs vertical tip deflection as a function of wetted length is depicted in Figure 20d.

**Figure 21:**
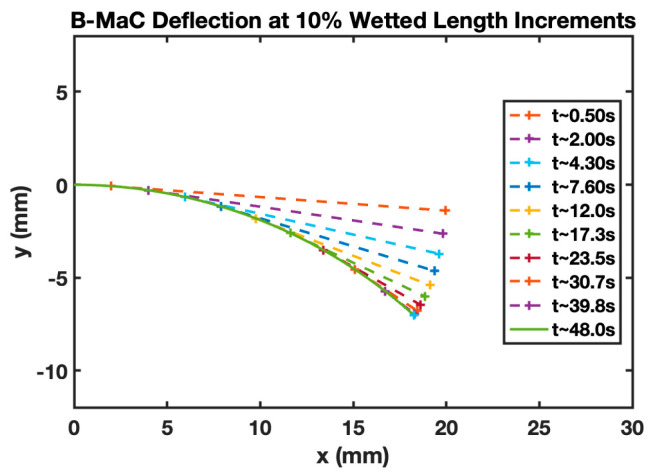
The B-MaC wetted curvature vs. time for every 10% wetted length increment.

Figure 21 shows the B-MaC wetted curvature for different time instances. The LW parameters were determined experimentally for the purpose of validating the model. Including time for the deflection of the B-MaC gives the reader insight into the dynamic loading of the B-MaC; for each time, the wetted length increases by 10% until the B-MaC is fully wetted.

**Figure 22:**
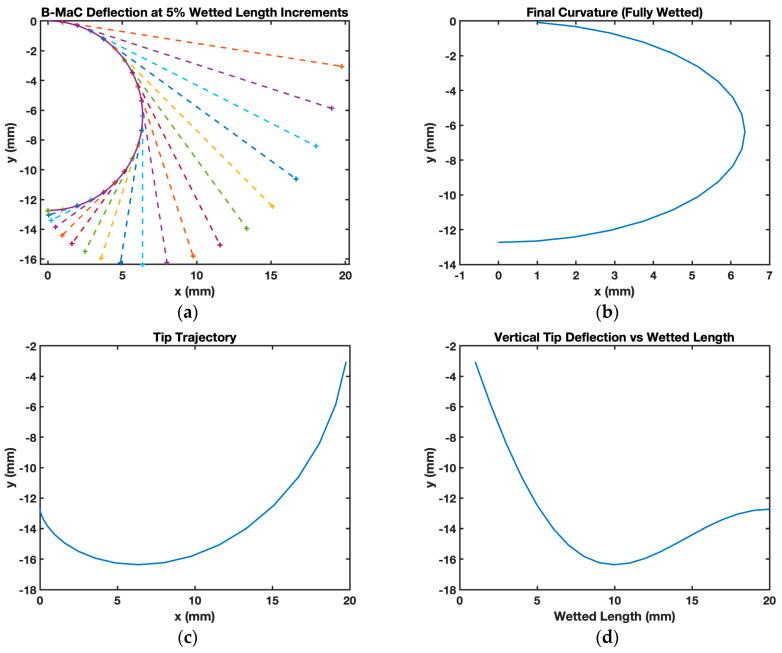
Matlab plots for the analytical model of a Whatman filter paper grade 41 Scotch tape B-MaC when the hygroscopic strain parameter is changed from 0.007 to 0.03. (**a**) The B-MaC wetted curvature and corresponding dry length for every 5% wetted length increment; (**b**) final curvature after full actuation; (**c**) tip trajectory after full actuation; (**d**) vertical tip deflection as a function of wetted length.

Next, the model was rerun using the same parameters, except for the hygroscopic strain, which was changed from 0.007 to 0.03 to simulate a larger hygroscopic expansion in the paper. For this case, the model predicts the B-MaC to have a smaller radius of curvature (6.3 mm) and a larger maximum vertical tip deflection (16 mm). This was expected since the larger hygroscopic expansive strain results in a smaller radius of curvature, as seen in Figure 22a,b where the B-MaC bends into a semicircle.

### 4.2. Comparison between Experiment and Model

**Figure 23:**
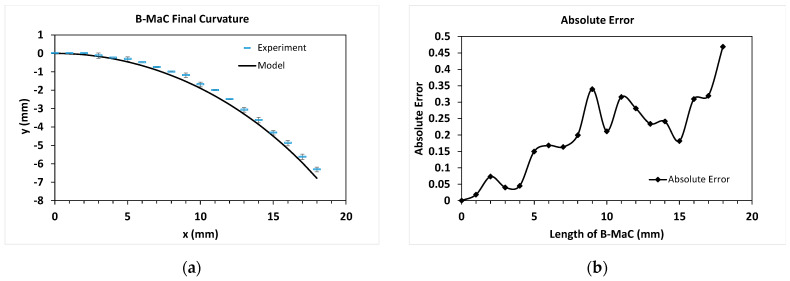
Comparison between the experiment and analytical model calculations. (**a**) Final curvature after full actuation. Measurements from the experiment are plotted as dashes with standard deviation. Calculated results from the model plotted as a solid line; (**b**) absolute error between the model and each data point from the experiment.

**Figure 24:**
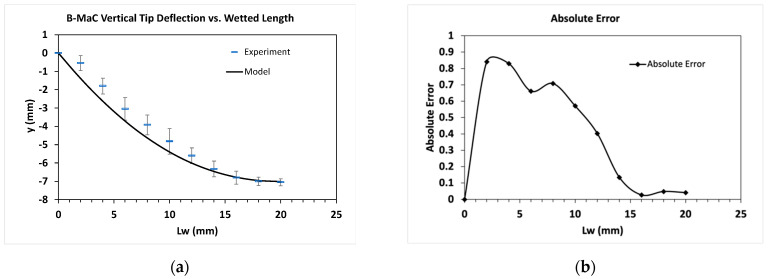
Comparison between the experiment and analytical model calculations. (**a**) Vertical tip deflection plotted as a function of wetted length. Measurements from the experiment are plotted as dashes with standard deviation. Calculated results from the model are plotted as a solid line. (**b**) the absolute error between the model and each data point from the experiment.

To verify the analytical model’s accuracy, comparisons were made to the empirical data collected from the experiment in Section 2.3. Data points from four B-MaC actuations were averaged and plotted as dashes with standard deviation in the final curvature plot in Figure 23a and the vertical tip deflection versus wetted length plot in Figure 24a. Results from the analytical model were plotted as a solid line. As seen in both figures, the model and the experimental measurements are in good agreement with each other. In Figure 24a, the model slightly overpredicts the curvature but captures the overall trend reasonably well. Similarly, in Figure 24a, the model slightly overpredicts the tip deflection at the start of actuation, but the overall trajectory is captured reasonably well.

The absolute errors, $\left|y_{experiment}-y_{model}\right|$, between the model and the discrete experimental data points, are shown in Figure 23b and Figure 24b. One explanation for the high relative error for some data points towards the middle of the cantilever can be attributed to the fact that the experimental data points had to be measured by eye due to equipment limitations in the lab. This restricted our resolution during data collection, forcing us to estimate each measurement to the nearest quarter millimeter since it was impossible to visually discern smaller increments. Therefore, rounding errors compounded with the inherent inaccuracy of visual measurements all likely contribute to the overall error, which is especially high at the start of actuation. Another explanation for the error could stem from the parameter values selected for the analytical model, particularly Young’s moduli and hygroscopic strain, which were determined experimentally. Therefore, it is possible their values were not fully accurate and could have introduced errors in the model output.

### 4.3. Model Limitation

A limitation of the presented analytical model is that it does not take gravity into consideration. As seen in Section 2.2.4, the bending effects due to the weight of the absorbed fluid can alter curvature depending on B-MaC orientation, support position, and overall length. Gravitational effects are especially prominent in longer B-MaCs, where overall bending is driven more by self-weight than the strain mismatch. For simplicity, the model was built on the assumption that gravitational effects would not be significant. Therefore, the model accuracy is likely to decrease when trying to describe relatively long B-MaCs, where the bending moments due to gravity are dominant. Further experimentation is required to determine for which length, and other B-MaC parameter combinations this model is appropriate.

The novelty of a paper-based cantilever and thermo-mechanical model for a bi-metal thermostat lies in the use of paper as the material for the cantilever and the thermo-mechanical model for analyzing the behavior of the bi-metal thermostat. The development of thermo-mechanics models specifically tailored to the properties of the paper-based cantilever allows for a more accurate prediction of the behavior of the bi-metal thermostat. This model considers the unique properties of the paper, such as its hygro-expansion actuation strain and its sensitivity to changes in moisture content.

## 5. Conclusions

In the present work, multiple B-MaC experiments were performed, and the bending behavior of fluidically activated paper-based bi-material cantilevers of different paper and tape types was investigated. Observations from these experiments were used to develop an analytical model describing the bending behavior of a B-MaC. The analytical model was compared to experimental data, and a good match was obtained. The quantitative results confirmed the validity of the analytical model in describing the curvature and tip deflection of a 20 × 4 mm Whatman 41-Scotch tape B-MaC. To better determine the limiting parameters and gauge the accuracy of the proposed model, further experimentation has to be conducted. Furthermore, future models need to incorporate dynamic fluid flow and be able to calculate the location of all segments of the cantilever as a function of time. Doing so will significantly aid in the optimization of future B-MaC designs for use within a μPAD.

## Data Availability

Data is contained within the article. Additional data not presented in this article is available on request from the corresponding author.
